# Delayed inactivation of TRPC6 as a determinative characteristic of FSGS-associated variants

**DOI:** 10.1016/j.jbc.2025.110256

**Published:** 2025-05-21

**Authors:** Ryo Okada, Reiko Sakaguchi, Tatsuya Komaki, Ryu Nonaka, Onur K. Polat, Takanori Kihara, Katsuhiko Asanuma, Takeshi Yamamoto, Yoshitaka Isaka, Yasuo Mori, Masayuki X. Mori

**Affiliations:** 1 Human Information and Life Sciences, School of Health Sciences, University of Occupational and Environmental Health, Fukuoka, Japan; 2 Laboratory of Biomaterials and Chemistry, School of Medicine, University of Occupational and Environmental Health, Fukuoka, Japan; 3 Faculty of Environmental Engineering, Department of Life and Environment Engineering, The University of Kitakyushu, Fukuoka, Japan; 4 Sealy Institute of Drug Discovery and Department of Neurobiology, University of Texas Medical Branch, Galveston, Texas, USA; 5 Department of Nephrology, Graduate School of Medicine, Chiba University, Chiba, Japan; 6 Department of Nephrology, Graduate School of Medicine, Osaka University, Osaka, Japan; 7 Department of Synthetic Chemistry and Biological Chemistry, Graduate School of Engineering, Kyoto University, Kyoto, Japan

**Keywords:** transient receptor potential channels (TRP channels), podocyte, kidney, FSGS, calcium signaling, phosphatidylinositol signaling, F-actin, patch-clamp, CRISPR/Cas

## Abstract

Transient receptor potential canonical 6 (TRPC6) is a receptor-operated nonspecific cation channel. To date, more than 30 TRPC6 variants have been reported to focal segmental glomerulosclerosis (FSGS), which can present from infancy to adulthood and is characterized by proteinuria and often nephrotic syndrome leading to kidney failure. These variants may exhibit gain-of-function (*e.g.* K874X) or loss-of-function (*e.g.* L395A, G757D) phenotypes, making the role of TRPC6 in FSGS controversial. Here, we characterized Ca^2+^-dependent inactivation (CDI) of TRPC6 after the receptor activation and found that >85% of TRPC6 variants exhibit delayed CDI. Thus, prolonged TRPC6 channel opening due to impaired inactivation may be a common feature of FSGS-associated variants. This effect was confirmed in immortalized mouse podocytes (MPC-5) in which the coiled-coil (CC) domain was deleted from the channel (C6_Δ_CC). Podocytes expressing C6_Δ_CC exhibited delayed CDI and increased basal Ca^2+^ levels as well as disruption of the F-actin cytoskeleton. Moreover, transcriptomic data from C6_Δ_CC-expressing podocytes showed weak expression of the podocyte markers Synpo and Magi2. These results indicate that CDI of TRPC6 is critical for maintaining proper podocyte function. Notably, we observed a correlation between the magnitude of the prolongation of TRPC6 channel activity and the age diagnosed with FSGS. Our findings thus demonstrate that delayed inactivation due to lack of CDI is a determinative characteristic of FSGS-associated TRPC6 variants, affecting both the structure and the function of glomerular podocytes.

Transient receptor potential canonical or classical (TRPC1-7) channels are mammalian homologs of the *Drosophila* TRP channel and members of TRP superfamily (1, 2). TRPCs are generally linked to metabotropic G-protein-coupled receptors and activated upon phospholipase C-catalyzed hydrolysis of phosphatidylinositol 4,5-bisphosphate (PIP_2_). Several critical mechanisms by which gating of these channels is modulated have been reported (3, 4). For instance, all TRPC channel isoforms appear to be positively regulated through direct association with PIP_2_ (5, 6). Phosphorylation by protein kinase A (PKA) or PKG suppresses TRPC4, TRPC5, and TRPC6 channel activity (7, 8). Intracellular Ca^2+^ potentiates TRPC5 channel activity (9), while Ca^2+^-bound calmodulin (CaM) directly inhibits TRPC3, TRPC4, and TRPC6 channels (10, 11, 12). Although these studies suggest that TRPC channels are key effectors of cellular adaptation to environmental changes, it remains unknown how these regulatory mechanisms contribute to human pathophysiology.

The TRPC6 channel is known to be expressed in renal glomerular podocytes in cases of familial focal segmental glomerulosclerosis (FSGS) (13). Over the past 10 years, nearly 30 TRPC6 variants have been reported in patients with FSGS of varying ages (14, 15, 16, 17, 18, 19, 20, 21, 22, 23, 24, 25, 26, 27, 28, 29, 30, 31, 32). Often, FSGS-associated TRPC6 variants exhibit “gain-of-function” phenotypes (14, 15, 16), although some of the variants, including L395A and G757D, reportedly cause a “loss-of-function” (29, 32). Due to their complex characteristics, the fundamental regulatory mechanisms governing the activity of these TRPC6 variants remain unclear, and a comprehensive understanding of the damage they cause to podocytes is still lacking.

We previously proposed that CaM-mediated negative regulation of TRPC6 induced by increases in the intracellular Ca^2+^ concentration ([Ca^2+^]_i_)—*i.e.,* Ca^2+^-dependent inactivation (CDI) of the channel—is impaired in some FSGS-associated TRPC6 variants (33). This negative regulation, which can be recorded under low Ca^2+^-buffering conditions, is mediated by the coiled-coil (CC) domain and the Ca^2+^-bound CaM-bridging complex, and deletion or mutation of the CC domain delays inactivation. However, structural studies have also shown the close proximity of the C-terminal CC to the N-terminal ankyrin-repeat domain (ARD), suggesting both of these regions may contribute to the negative regulation of TRPC6 (34). Consistent with that idea, FSGS-related variants are often found within the ARD.

Pathophysiological variant mutations are found in the *TRPC6* gene; the ages of the patients at diagnosis of FSGS are quite broad, from infants to adults (mean ± SD is 20.4 ± 17.0 years). We hypothesized that the degree to which the negative regulation of TRPC6 is impaired differs among the FSGS variants and accounts for the broad range of the ages of disease onset. To test that idea, in the present study, we characterized the phenotypes of FSGS-associated variants and determined the level of negative regulation as a gauge of the linkage to FSGS.

## Results

### Functional analysis of the inactivation in the TRPC6 channel

**Figure 1:**
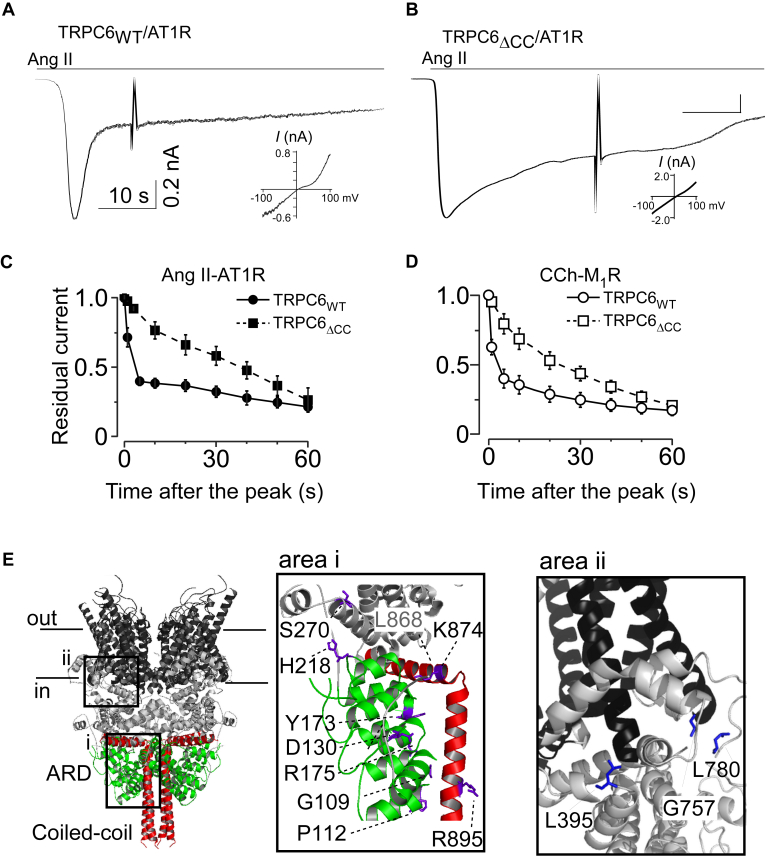
**Comparison of TRPC6 operating receptors on inactivation of TRPC6 and structural mapping of FSGS-associated variants**. *A* and *B*, representative currents recorded from HEK293 cells expressing (*A*) human TRPC6_WT_ and (*B*) the CC domain deletion (TRPC6_ΔCC_) variants. Currents were evoked by Ang II (*gray top bar*). Sharp noises in the current traces indicate a ramp pulse from −100 mV to +100 mV (insets) which represents a typical doubly rectifying *I*-*V* curve of TRPC6 currents. *C*, fractions of residual currents plotted against time after the peak. *Black circles and boxes* indicate data from TRPC6_WT_ or TRPC6_ΔCC_ with AT1R-transfected cells, respectively. *D*, inactivation plots constructed by plotting residual currents for TRPC6_WT_ and TRPC6_ΔCC_ with M_1_R-transfected cells are shown as *open circles and boxes*, respectively. *E*, ribbon diagram structure of human TRPC6 was modified from PDB 6UZ8 (34). Transmembrane, ankyrin-repeat domain (ARD), and the CC domain are colored in *black*, *red*, and *green*, respectively. The *right panels* (area i and area ii) show a zoomed view of the N-terminal ARD/the C-terminal CC domain and the juxta-membrane domain, respectively. Amino acid residues associated with FSGS when mutated are highlighted as *blue* sticks.

We first compared the inactivation of TRPC6-derived currents mediated *via* several different receptors. Angiotensin Ⅱ (Ang II) and its receptor (AT1R) are critical contributors to glomerular disease (35). AT1Rs reportedly exhibit rapid desensitization kinetics (36), but the effect of Ang II-AT1R activation on TRPC6 channel inactivation has not been tested or compared with activation of conventional muscarinic acetylcholine type 1 receptors (M_1_Rs), which desensitize more slowly. As shown in Figure 1*A*, Ang II-AT1R-operated TRPC6 currents inactivate after a peak. The degree of that inactivation, which was evaluated based on the residual currents after the peak, was nearly identical to the inactivation degree of carbachol-M_1_R-operated currents (Fig. 1, *C* and *D*). In addition, the CC domain deletion variant (TRPC6_ΔCC_) showed similarly delayed inactivation after AT1R or M_1_R stimulation (Fig. 1, *B*–*D*). This suggests receptor-operated TRPC6 channel inactivation is equally induced, despite the different desensitization kinetics of AT1Rs and M_1_Rs.

**Figure 2:**
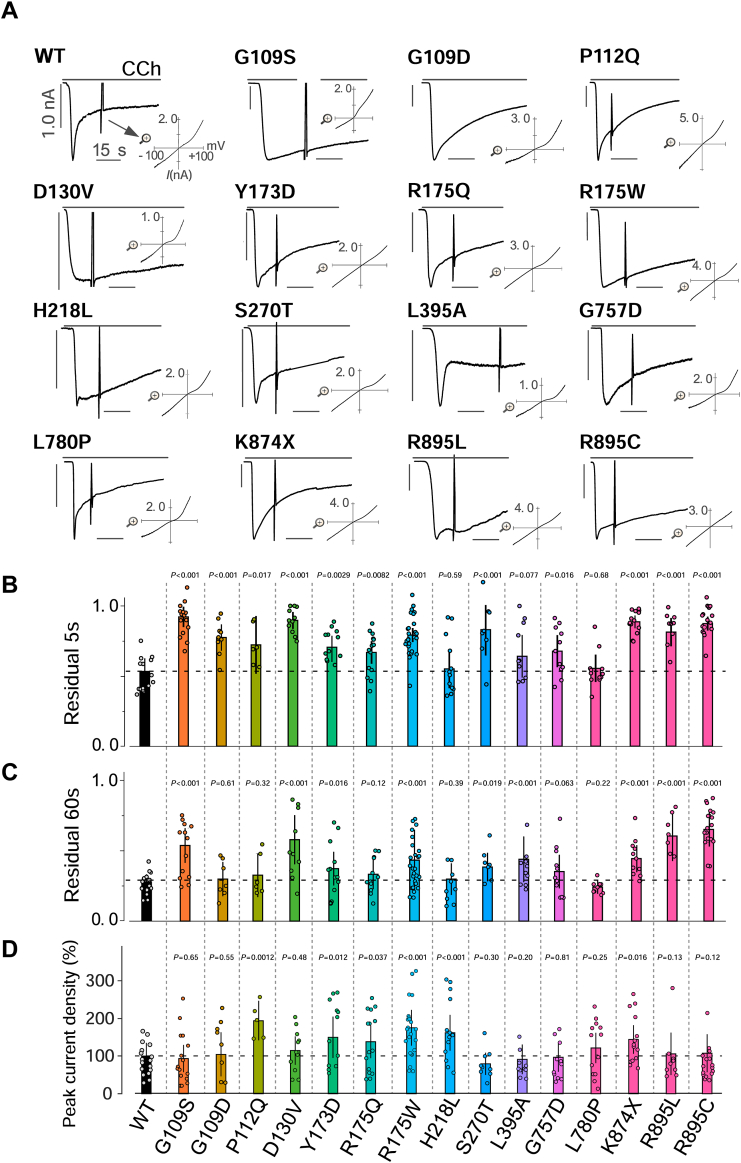
**Gain-of-function phenotypes in FSGS-associated TRPC6 variants.***A*, representative currents recorded from HEK293 cells expressing TRPC6_WT_ and FSGS-associated variants. Currents were evoked by CCh. *B* and *C*, summary of residual currents after 5 s and 60 s from the peak. The columns and bars represent the mean and SD, respectively (n > 5). *p* values that are significantly different from the wild-type receptor value were calculated using Student’s *t* test. *p* values are denoted above bars. Individual data points indicate individual cells measured. *D*, summary of peak current densities (pA/pF) of FSGS-associated TRPC6 variants. The *columns* and *bars* represent the means of current densities which were normalized by the mean of TRPC6_WT_ currents and SD, respectively. *p* values that are significantly different from the wild-type receptor value were calculated using Student’s *t* test. *p* values are denoted above *bars*. Individual data points indicate individual cells measured.

We then tested 15 FSGS-associated TRPC6 variants, of which 12 were located at the interface of the ARD and CC domain (Fig. 1*E*, area i, Fig. S1), while three were close to the transmembrane region (Fig. 1*E*, area ii, Fig. S1). In earlier studies, gain of function was assessed by measuring the peak-current amplitude or the maximum [Ca^2+^]_i_ (14, 15). Correspondingly, loss of function was assessed based on the reduction of the peak currents or the maximum [Ca^2+^]_i_ (29, 32). As summarized in Figure 2*D*, among the 15 TRPC6 variants tested, 6 (40%) exhibited higher peak current density than wild-type (WT) TRPC6, with a rank order of P112Q > R175W > H218L > Y173D> K874X > R175Q. The remaining nine FSGS variants, including L395A and G757D, did not increase peak current density. We confirmed the expression levels of channel proteins in FSGS variants that exhibited either high or non-alternating peak current density. The expression levels among wild-type and variants assessed by Western blotting showed no statistical difference (Fig. S2). This blotting result suggested that the protein expression differences in the FSGS variants are negligible in affecting the size of current densities.

Despite this variation, delayed inactivation was observed with most of the FSGS variants (Fig. 2, *A*–*C*). Twelve of the FSGS-associated variants (80%) exhibited significantly slowed inactivation of residual currents at 5 s (Fig. 2*B*). Even during the late phase of the residual current (60 s after the peak), nine (60%) variants still exhibited slower inactivation than the WT TRPC6 (Fig. 2*C*). Among those variants, L395A showed significant slowing of inactivation only during the late phase. In total, 13 of the 15 variants (86.7%) exhibited delayed inactivation within 60 s. Thus, the number of variants exhibiting delayed inactivation was more than twice that of those showing increased peak current density. These findings support the idea that delayed inactivation is a common feature of FSGS-associated variants.

Based on our electrophysiological analysis, FSGS-related TRPC6 variants were categorized into four groups: (1) delayed inactivation without amplification of the peak current density (G109S, G109D, D130V, S270T, L395A, G757D, R895C and R895L; 8/15 = 53%); (2) delayed inactivation with increased peak current density (P112Q, Y173D, R175Q, R175W and K874X; 5/15 = 33%); (3) increased peak current density with no alteration of the inactivation (H218L; 1/15 = 7%); and (4) no significant differences from the WT currents (L780P; 1/15 = 7%). Overall, 86.7% of FSGS-related TRPC6 variants [groups (1, 2)] exhibited prolonged channel activity, the underlying mechanism of which was delayed inactivation.

### Disruption of CDI in FSGS-associated TRPC6

**Figure 3:**
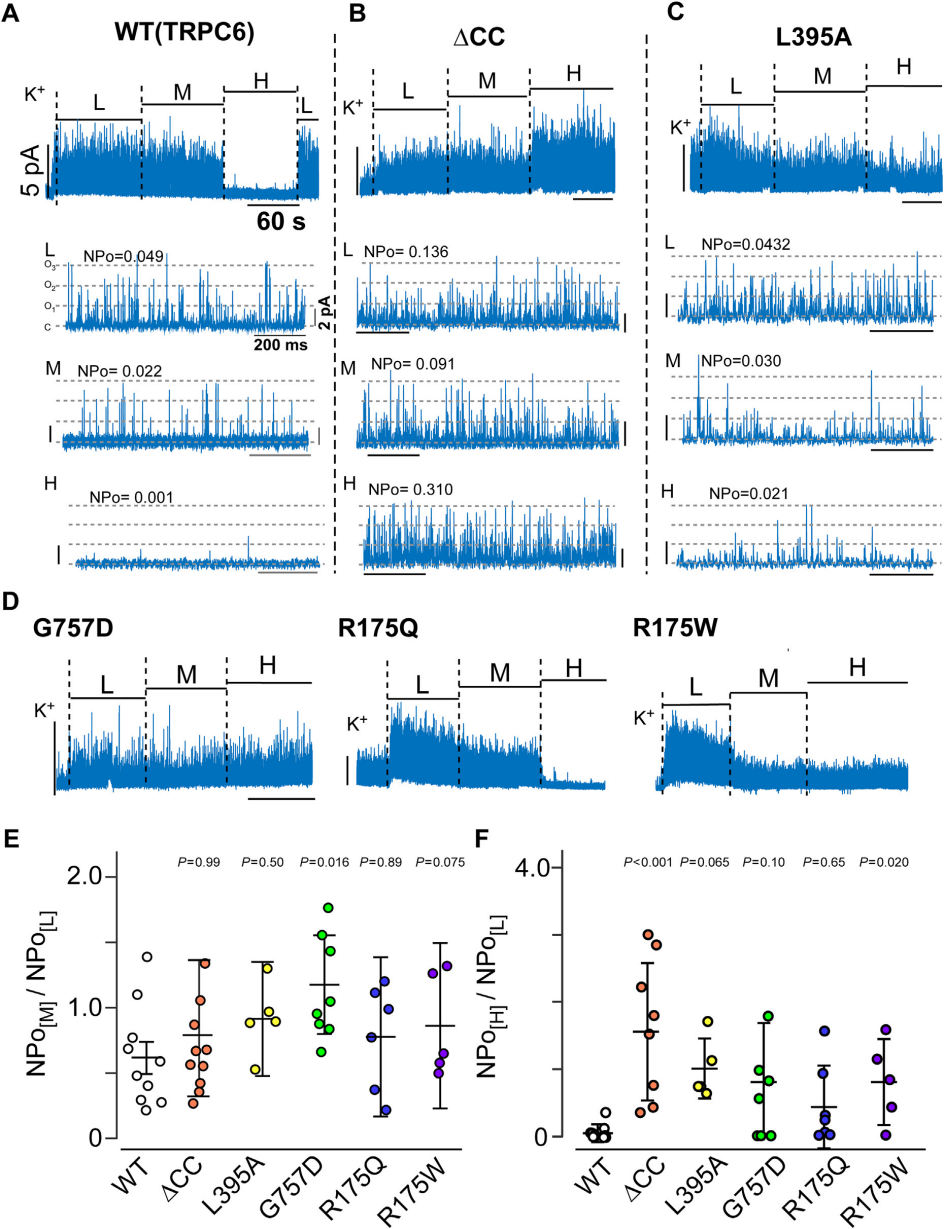
**The i/o recording of coiled-coil deletion and FSGS-associated TRPC6 variants**. *A–C*, Representative inside-out recordings are shown in the *upper panels*. Intracellular Ca^2+^ concentrations were step-wisely altered from L (0.1 μM), M (0.6 μM), H (1.2 mM). (*A*) TRPC6_WT_, (*B*)ΔCC, and (*C*) area ii variant L395A. Enlarged traces and nominal open probability (NPo) *versus* [Ca^2+^]_i_ are shown in the respective *bottom panels*. *C*; closed state, O_1_-O_3_; open states. *D*, representative i/o recordings from area ii variant G757D, area i variants R175Q and R175W, respectively. (*E, F*) Summary of the i/o recordings of TRPC6_WT_ (*open circles*), ΔCC (*orange circles*), L395A (*yellow circles*), G757D (*green circles*), R175Q (*blue circles*), and R175W (*purple circles*) are depicted as a plot of NPo. Normalized NPo was obtained by dividing (*E*) NPo at M (0.6 μM) and (*F*) NPo at H (1.2 mM) by NPo at L (0.1 μM), respectively. The bars represent the mean and SD. *p* values that are significantly different from the wild-type receptor value were calculated using one-way ANOVA with Dunnett’s *post hoc* test. Individual data points indicate the results of individual i/o recording.

We previously reported that the delayed inactivation of FSGS-associated TRPC6 variants was related to their insensitivity to [Ca^2+^]_i_ (33). To verify this finding, we made inside-out (i/o) patch recordings while exposing different variants to stepwise increments in [Ca^2+^]_i_. With the WT channel, the open probability gradually decreased in response to the increments in [Ca^2+^]_i_ (Fig. 3*A*). Compared to this Ca^2+^-dependent profile, the TRPC6 CC domain deletion variant (ΔCC) and the R175W, L395A, and G757D FSGS-associated variants showed less sensitivity to [Ca^2+^]_i_ (Fig. 3, *B*–*F*). This suggests the channel activity of FSGS-associated variants is less sensitive to increasing [Ca^2+^]_i_. Intriguingly, the R175W variant was even less sensitive to [Ca^2+^]_i_ than the R175Q variant (Fig. 3*D*). Because the R175W variant, which showed remarkably delayed inactivation (Fig. 2), has been identified in infants diagnosed with FSGS (21, 27), we speculate that the magnitude of the inactivation delay caused by insensitivity to [Ca^2+^]_i_ may correlate with the onset age of FSGS. We address this possibility further in the Discussion section (Fig. 7).

**Figure 4:**
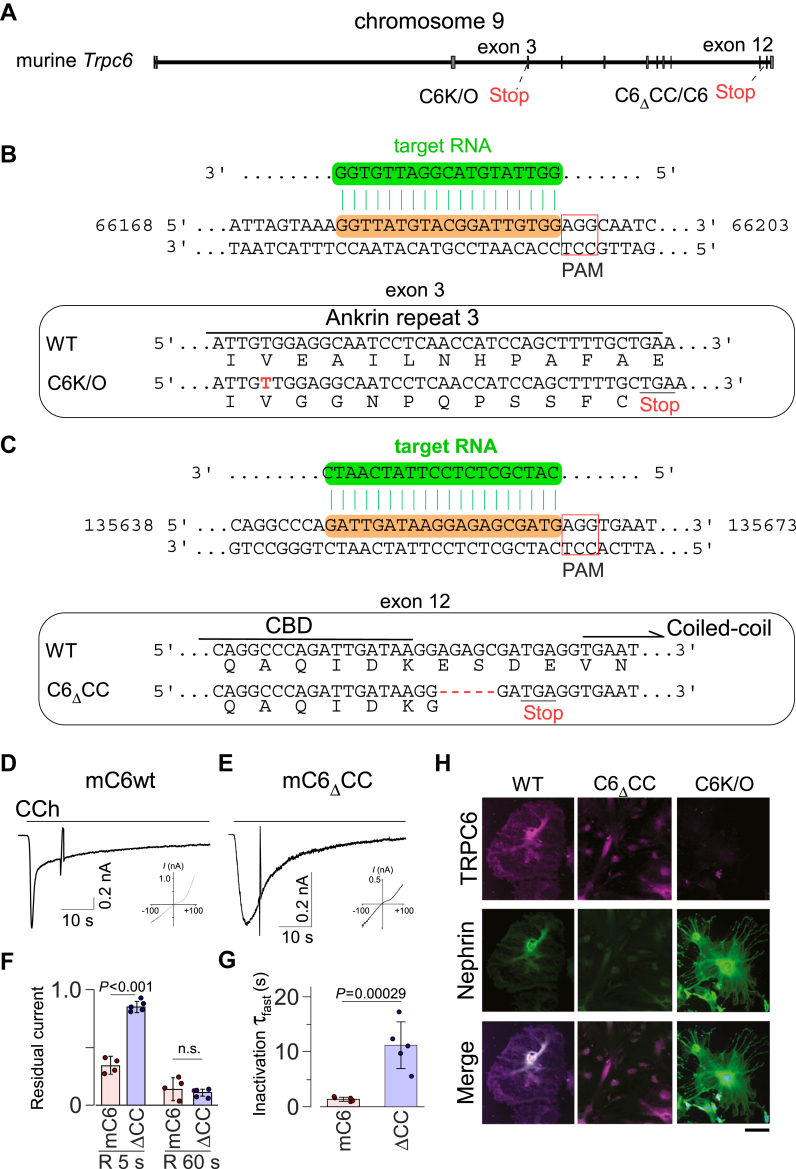
**Generation of TRPC6 knock-out and its coiled-coil deletion podocytes by CRISPR-Cas9**. *A*, schematic view of the *TRPC6* gene coded by 13 exons. The screening of mutation by PCR sequencing resulted in a flame-shift stop codon of the single base pair insertion in exon 3 and the five base pair deletion in exon 12 that generated C6K/O and C6_Δ_CC, respectively. *B* and *C*, sequences in exon 3 or exon 12 of the murine *TRPC6* gene were chosen as target sequences (highlighted in *orange*) for Cas9 enzyme. For generation of C6K/O, sequencing analysis of exon 3 confirmed single nucleotide insertion at 66,172 (NCBI Gene ID:22068) which resulted in partial protein at 186 a.a. For generation of C6_Δ_CC, sequencing analysis of exon 12 confirmed deletion of five nucleotides at 135,657 which resulted in entire deletion of C-terminal CC domain. *D* and *E*, representative currents recorded from HEK293 cells expressing mouse TRPC6_WT_ and _Δ_CC variants. *F*, summary of residual currents after 5 s and 60 s from the peak. The columns and bars represent the mean and SD, respectively. Results for WT and _Δ_CC were compared using Student’s *t* test. Individual data points indicate individual cells measured. *G*, summaries of the fast inactivation kinetics. The fitting equation was as follows, *I*_*inactivation*_(*t*) = *A*_*1*_*exp(-t/τ*_*fast*_*)* + *A*_*2*_*exp(-t/τ*_*slow*_*)* + *C,* where *I* is current, *t* is time (sec); *A*_*1*_ and *A*_*2*_ are initial quantities; *τ*_*fast*_ and *τ*_*slow*_ are time constants; *C* is an offset, respectively. The *columns* and *bars* represent the mean and SD, respectively. *p* values were calculated using Student’s *t* test. Individual data points indicate individual cells measured. *H*, immunofluorescence staining of wild-type MPC-5 (WT), C6_Δ_CC, and C6K/O cells with antibodies against TRPC6 (shown as *magenta pseudocolor*) and nephrin (*green*). C6K/O cells showed little or no staining with TRPC6 antibody but robust signal with nephrin antibody.

**Figure 5:**
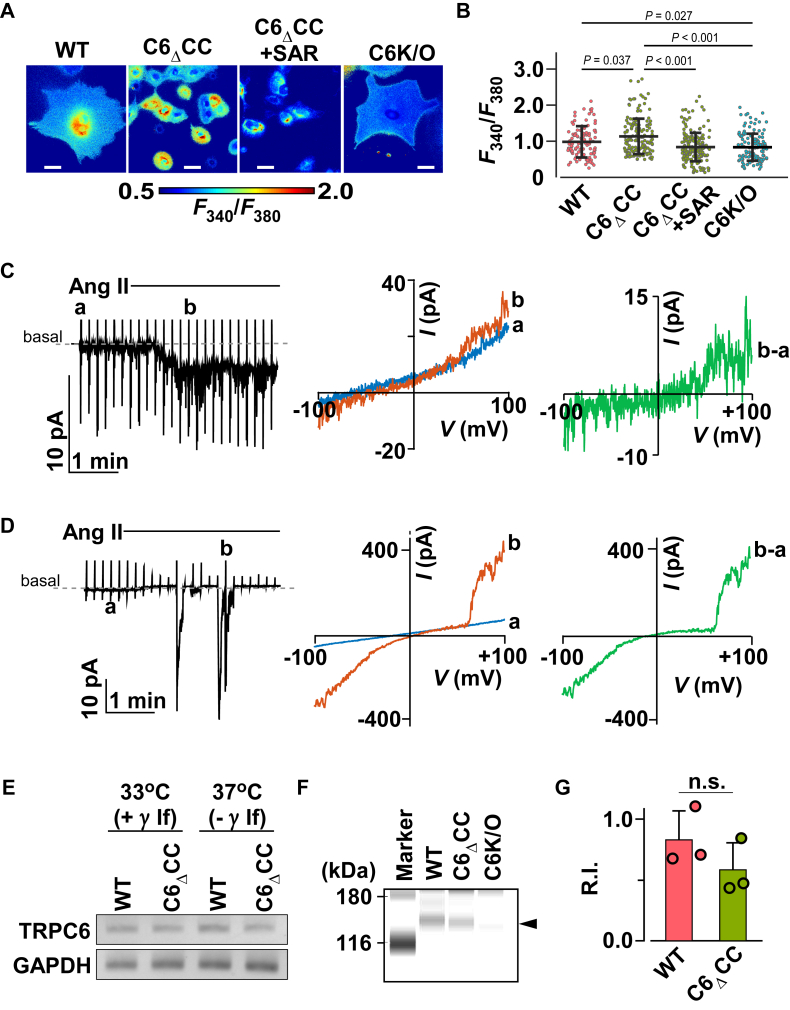
**Gain-of-function phenotype of the coiled-coil deletion podocytes and contribution of the channel surface expressions**. *A*, intracellular Ca^2+^ imaging with Fura-2AM loaded MPC-5 cells. The *white bars* indicate 50 μm. *B*, summary of basal [Ca^2+^]_i_. The bars depict Fura-2 ratios (*F*_340_/*F*_380_). To evaluate the effect of TRPC6 specific inhibitor SAR-7334, C6_Δ_CC cells were pretreated 3 μM of the inhibitor for 30 min before the analysis. The *bars* represent the mean and SD. Results were compared using one-way ANOVA with Tukey’s *post hoc* comparison tests. Individual data points indicate individual cells examined. More than 50 cells were calculated in each group. *C and D*, whole-cell recordings from (*C*) WT and (*D*) C6_Δ_CC cells (*D*). A representative trace of whole-cell currents induced by 1 μM Ang II (*leftpanels*). The currents were recorded at a holding potential of −50 mV. Corresponding *I*-*V* relationships (from −100 to +100 mv) at time points a and b (*middlepanels*). The right panels show *I*-*V* relationships for the Ang II-induced TRPC6-like currents obtained by subtraction of the trace in the middle panels. *E*, mRNA expression levels in MPC-5 cells. *F*, surface biotinylation assay using anti-TRPC6 antibody. Arrowhead indicates a band of TRPC6. *G*, the statistical analysis is given for the surface biotinylation assay. To minimize experimental errors between individual runs, the signal intensity corresponding to TRPC6 was normalized by a biotinylated molecular marker (116 kDa). The *columns* and *bars* are mean ± SD, respectively. The uncropped image is shown in Fig. S7.

**Figure 6:**
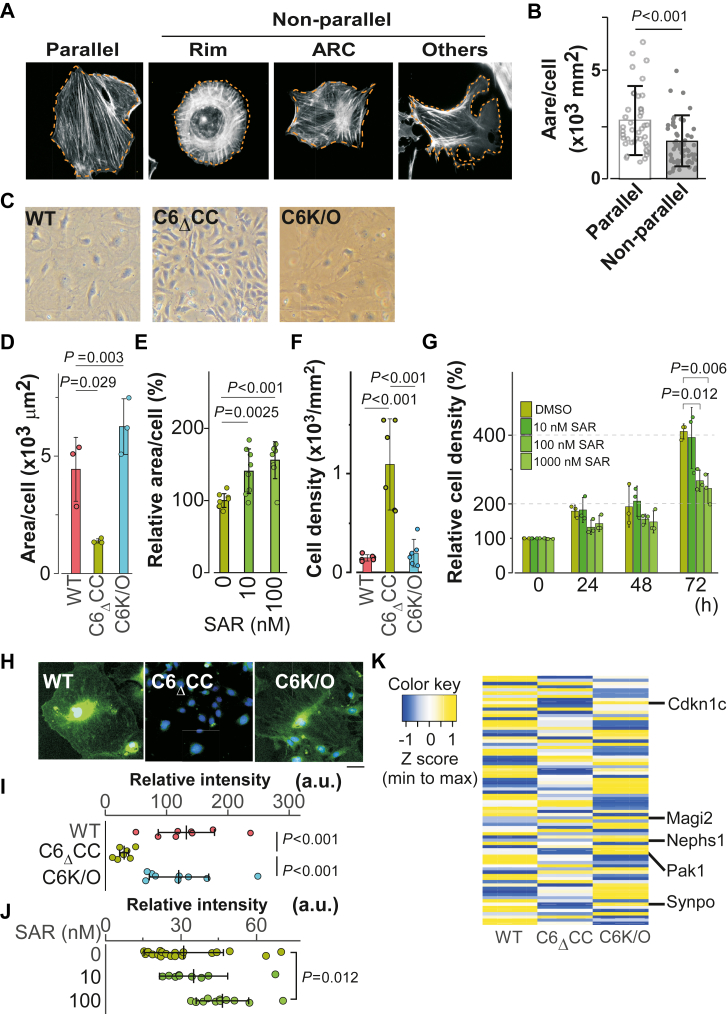
**Deletion of coiled-coil in TRPC6 affects cell morphology and differentiation**. *A*, unbiased evaluation of cell morphology based on the cell sizes. Panels show F-actin configurations of parallel stress fibers “parallel,” rim like structure “rim”, presence of actin-rich center (“ARC”), and non-definable actin structure (“others”). Individual cell areas were calculated as shown in *orange dotted lines*. *B*, graphs show quantified results of cell sizes in healthy parallel fiber type and damaged nonparallel type. The *columns* and *bars* represent the mean and SD, respectively, here and throughout. *p* value was calculated using Student’s *t* test. Individual data points indicate individual cells examined. *C*, Bright-field images of MPC-5 cells. *D*, Single-cell area (area/cell). Results were compared using one-way ANOVA with Tukey’s *post hoc* comparison tests. Individual data points indicate the area of each cell in an individual image. *E*, relative single cell area of C6_Δ_CC cells treated with SAR-7334. *p* values that are significantly different from the cells without SAR-7334 treatment were calculated using one-way ANOVA with Dunnett’s *post hoc* test. *F*, cell density after 2 weeks differentiation was significantly increased in C6_Δ_CC cells. Results were compared using one-way ANOVA with Tukey’s *post hoc* comparison tests. Individual data points indicate the number of cells in an individual image (1200 μm × 670 μm). *G*, time courses of C6_Δ_CC cell density with the treatment of SAR-7334. The mean of initial plating cell density was set to baseline. *p* values that are significantly different from the cells without SAR-7334 treatment at 72 h were calculated using one-way ANOVA with Dunnett’s *post hoc* test. *H*, immunofluorescence staining of MPC-5 cells with synaptopodin antibody (*green*) and DAPI staining (*blue*). The scale bar indicates 50 μm. All images are representative of six replicates. *I*, quantitative analysis of synaptopodin immunofluorescence intensity. Results were compared using one-way ANOVA with Tukey’s *post hoc* comparison tests. Individual data points were from each cell. *J*, the TRPC6 inhibitor restored expression of synaptopodin in C6_Δ_CC cells. Individual data points were from each cell. *p* values were calculated using one-way ANOVA with Dunnett’s *post hoc* test. The *bars* in (*I*) and (*J*) represent the mean and SD. *K*, heat map of the podocyte-specific essential genes summarized by Lu *et al.* (68), in the comparison of WT (*left*), C6_Δ_CC (*middle*), C6K/O (*right*) cells. The color intensity represents column *Z* score, with *yellow* indicating higher expression and *blue* indicating lower expression compared with the average gene expression level of three cell lines. The TPM values of genes in this Heat map were listed in Fig. S4.

**Figure 7:**
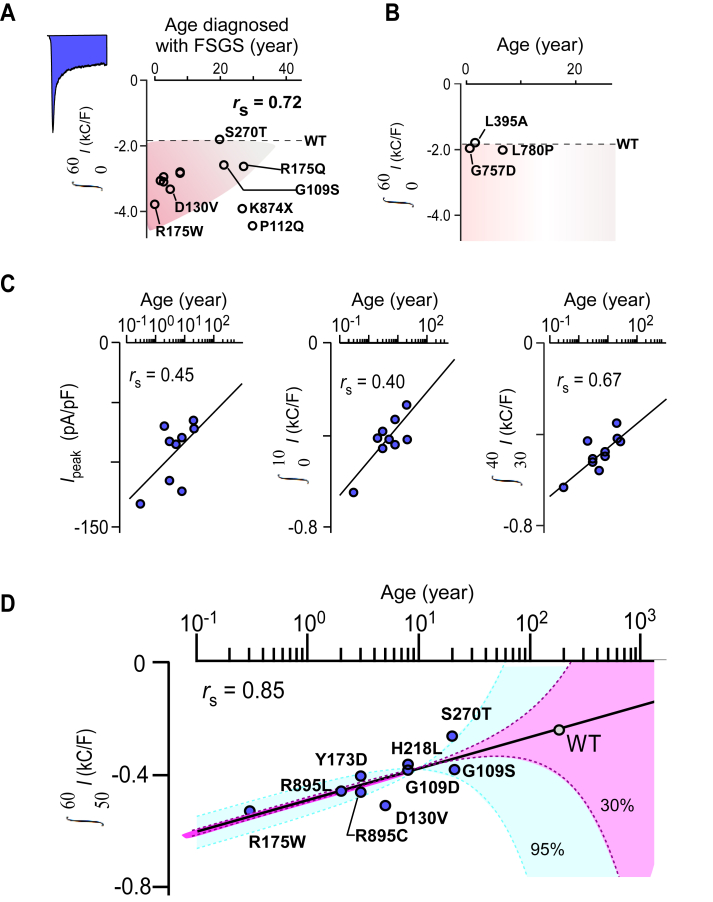
**Correlation of integrated current densities of TRPC6 variants and the age when diagnosed as FSGS**. *A*, the integrated current densities from 0 to 60 s after CCh stimulation of area i TRPC6 variants were plotted against the ages diagnosed with FSGS (years). The *bluepainted* area counted for the integrated current density. The *horizontal dash line* indicated the integrated current density of wild-type TRPC6. *B*, the integrated current densities from 0 to 60 s in the area ii mutants are plotted. *C*, the peak current density and the integrated current densities from 0 to 10 s, from 30 to 40 s in the area i variants were plotted. *r*_s_ denotes Spearman's rank correlation coefficient. *D*, mean values and 95% or 60% confidence intervals (*blue* or *pink*) of the integrated current densities from 50 to 60 s estimated for the ages diagnosed with FSGS are plotted. Data points were fitted by the non-linear regression analysis (*black line*). This correlation suggests that the onset age for FSGS in a person with the WT TRPC6 channel ranges from 125 to 235 years (with a median age of 171 years).

### Establishment of a podocyte cell line lacking TRPC6 CDI

The cellular effects of the delayed TRPC6 inactivation due to a lack of CDI were examined in MPC-5 cells, a murine-derived podocyte cell line. Using the CRISPR-Cas9 system, we generated a TRPC6 gene lacking its CC domain (clone C6_Δ_CC, Fig. 4, *A* and *C*). The coiled-coil lacking murine TRPC6 (mC6_Δ_CC) exhibits sustained residual currents (Fig. 4, *D*–*F*). To gain further insight into the inactivation, we proceeded to the kinetic analysis of the decay currents. The inactivation kinetics were double phasic, showing a fast phase followed by a slow phase (Fig. 4*D*). The fast inactivation kinetics (*τ-*fast) of mC6_Δ_CC were 8-fold slower than the mC6_WT_ currents (1.35 ± 0.37 s with mC6wt and 11.1 ± 4.22 s with mC6_Δ_CC, Fig. 4*G*). Contrarily, the slower inactivation (*τ-*slow) did not show any differences (45.9 ± 3.4 s with mC6wt and 41.6 ± 7.4 s mC6_Δ_CC). For the comparison, we also generated a clone carrying a stop codon at exon 3 (clone C6K/O, Fig. 4, *A* and *B*). Immunostaining showed TRPC6 to be distributed in the plasma membrane and perinuclear region in both WT MPC-5 cells and the C6_Δ_CC cells, but not in the C6K/O cells (Fig. 4*H*). In addition, colocalization with nephrin, which is the central component of slit diaphragm and known to bind TRPC6 (37), did not differ between WT and C6_Δ_CC cells. Apparently, the localization of nephrin is unaffected by the deletion of the coiled-coil domain and disruption of the inactivation function. Notably, C6K/O cells exhibited especially robust nephrin-derived signals as well as growth of foot process-like structures. These phenotypes suggest that TRPC6 channel activity has a suppressive effect on nephrin expression in podocytes.

The functional cellular effect of deleting the CC domain from TRPC6 was assessed through Ca^2+^ imaging (38). The basal [Ca^2+^]_i_ in C6_Δ_CC cells was significantly higher than in WT cells (Fig. 5*A*). However, after pretreatment with TRPC6-specific inhibitor SAR-7334 (3 μM, for 30 min) (39), basal [Ca^2+^]_i_ in C6_Δ_CC cells was reduced nearly to the level seen in C6K/O cells (Fig. 5*B*). This suggests that the loss of CDI caused by CC deletion from TRPC6 accounts for the increased basal [Ca^2+^]_i_. Consistent with that idea, it was previously reported that basal [Ca^2+^]_i_ is upregulated in podocytes isolated from TRPC6 knockout mice (40). By contrast, basal [Ca^2+^]_i_ was significantly lower in C6K/O cells than in WT or C6_Δ_CC cells. These results suggest the functional cellular effect of eliminating TRPC6 in podocytes is not compensated for by expression of other TRPC isoforms (*e.g.* TRPC3) (41).

To evaluate receptor-operated TRPC6 currents in MPC-5 cells, we undertook whole-cell patch-clamp recordings by applying Ang II. Compared with WT cells, C6_Δ_CC cells exhibited extraordinarily large TRPC6 current amplitudes (Fig. 5, *C* and *D*). These functional assessments indicate that the responses of C6_Δ_CC channels are enhanced under both static basal conditions and upon receptor stimulation.

The delayed inactivation of TRPC6 currents may also induce irregular surface expression of TRPC6(42). After determining that expression of TRPC6 mRNA in the C6_Δ_CC cells did not differ from that in WT cells (Fig. 5*E*), TRPC6 expression on the plasma membrane was examined using surface biotinylation assays. The results showed that endogenous surface expression of TRPC6 channels was indistinguishable between WT and C6_Δ_CC cells (Fig. 5, *F* and *G*). It thus appears that the CC domain deletion from TRPC6 in C6_Δ_CC cells causes a gain-of-function without significantly altering the protein’s surface expression.

### Impact of the CDI on podocyte morphology and differentiation

Cellular morphology is often dependent on an intact actin cytoskeleton. In podocytes, filamentous actin fibers (F-actin) play a critical role in the formation of foot processes and in filtration (42, 43). We therefore investigated whether the absence of TRPC6 CDI alters the structure of the actin cytoskeleton. To assess F-actin structure, we employed a non-scoring approach to evaluate cellular morphology while minimizing human bias and error. In applying this approach, we measured single cell area after rhodamine-phalloidin staining to evaluate the cell size and F-actin structure (Fig. 6*A*). Figure 6*B* illustrates that in a healthy cell, F-actin assumes a parallel structure that covers a larger area than is covered by unhealthy nonparallel F-actin structures. The nonparallel structures were categorized as ring-like (Rim), actin-rich centered (ARC), and uncategorized (Others). This result shows that measuring the single cell area is a potentially useful approach to estimating F-actin structure while minimizing human bias. To use this assay, MPC-5 cells were differentiated for 2 weeks on 24-well plates (Fig. 6*C*). Notably, the single cell area in C6_Δ_CC cells was reduced to 32% of that in WT cells (1390 ±110 *versus* 4440 ±1380 μm^2^/cell) (Fig. 6*D*). Treatment of this podocyte with SAR-7334 increased the single cell area around 150% compared to the C6_Δ_CC cells without the inhibitor (Fig. 6*E*). This indicates that CDI impairment of TRPC6 inactivation has a substantial effect on F-actin organization in C6_Δ_CC cells. In addition, we also realized that C6_Δ_CC cells lacking CDI grew to a density seven times higher than with WT cells (Fig. 6*F*). This proliferation activity was significantly suppressed by the treatment with the TRPC6-specific inhibitor (Fig. 6*G*). It is well-known that mature podocytes express specialized cytoskeletal-associated proteins such as synaptopodin (44). Therefore, to determine the maturation state of MPC-5 cells, we also assessed the levels synaptopodin expression (Fig. 6*H*). Immunofluorescence with anti-synaptopodin showed its expression to be markedly suppressed in C6_Δ_CC cells as compared with WT or C6K/O cells (Fig. 6*I*). This weak expression level of synaptopodin was partially restored by treatment of the TRPC6 inhibitor (Figs. 6*J*, S3). We further confirmed the expression level of synaptopodin by RNA-sequencing analysis. Comparison of the transcripts per million (TPM) in WT, C6K/O and C6_Δ_CC cells showed that expression of *Synpo* gene, which encodes synaptopodin, was diminished in C6_Δ_CC cells (Figs. 6*K*, S4). Moreover, other podocyte-specific genes were also suppressed in C6_Δ_CC cells. These included *Magi2*, *Pak1*, and *Nephs1* as well as *Cdkn1c*, which encodes p57^Kip2^, a potent negative regulator of podocyte proliferation (45). These results indicate that C6_Δ_CC cells are in a less differentiated state in which expression of podocyte-specific genes is suppressed, and this cellular state can be restored by the inhibition of cationic influx with TRPC6 inhibitor.

## Discussion

In this study, we categorized the phenotypes of FSGS-associated TRPC6 variants as follows: (1) delayed inactivation with no alteration in the peak current density (53%); (2) delayed inactivation with increased peak current density (33%); (3) increased peak current density with no delayed inactivation (7%); and (4) inactivation and peak current density did not differ from WT (7%). Thus, delayed inactivation appears to be a common feature affecting 86.7% of FSGS-associated variants. This suggests that electrophysiological analysis of inactivation and current density is a potentially useful tool for evaluating the linkage between the TRPC6 mutation and FSGS.

### Delayed inactivation is a common feature of FSGS-associated variants

TRPC channels, including TRPC6, are a group of receptor-operated nonselective cation channels (46, 47, 48). In podocytes, aberrant [Ca^2+^]_i_ signaling mediated by TRPC channels and store-operated Ca^2+^ entry has been shown to induce actin remodeling and lead to proteinuria (49, 50, 51). Previous reports also showed that several TRPC6 variants (*e.g.,* P112Q, K874X) induce a gain-of-function, as indicated by increases in peak current amplitudes and [Ca^2+^]_i_ responses (14, 15). However, other variants either did not affect (*e.g.* S270T) or decreased (*e.g.* L395A, G757D) current amplitudes (15, 32). This variability in the functional data highlights the fact that a functional analysis based on maximum responses does not provide a definitive means of discriminating between disease-associated mutations and mere polymorphisms in the *TRPC6* gene. The present study provides solid evidence that sustained currents related to delayed inactivation are a consistent feature of nearly all the analyzed FSGS-associated variants (Fig. 2).

*TRPC6* gene consists of 13 exons, and FSGS-related variants are abundant within exon2 (a.a. 87-315) and exon13 (a.a. 882-931), which encode the ARD (a.a. 97-242) and the CC domain (a.a. 881-921), respectively. These two intracellular domains are located at the N-terminus and C-terminus but are in contact each other (52, 53). We previously showed that the CC domain contributes to CDI (33); however, the function of the N-terminal ARD and the mechanism underlying the CDI was not elucidated. Our functional analysis of FSGS-associated variants in area i illustrated in Figure 1*E* (G109S, G109D, P112Q, D130V, Y173D, R175Q, R175W, S270T, K874X, R895L, R895C) clearly demonstrates a slowed inactivation. Even more intriguing is that the area ii mutants L395A and G757D, which are located in the juxta-membrane region (Fig. 2*E*), also exhibit slower inactivation. Given that a large majority of the FSGS-associated variants exhibit delayed inactivation, it seems unnecessary to categorize their phenotype as “gain-of-function” or “loss-of-function.” Instead, we suggest it is more useful to characterize their phenotype as “delayed inactivation.” Moreover, the cause of the inactivation delay can be attributed to the irregular [Ca^2+^]_i_ responses, as evidenced by the i/o patch recordings summarized in Figure 3. However, the area ii L780P variant showed no significant functional phenotype. According to the original report, L780P was found in a 7-year-old patient, but the same variant was found in healthy family members (the father and two brothers) (23). This suggests the emergence of FSGS may be caused by a combination of variants such as in the reported case associated with R68W mutation in *TRPC6* gene plus NPHS1 polymorphisms (54).

### Mechanistic insight into the delayed inactivation of FSGS-associated variants

Several factors, including phosphorylation, Ca^2+^, and PIP_2_, are reported to be critical regulators of TRPC channels (5, 55, 56). We previously demonstrated that the negative regulation by [Ca^2+^]_i_ was missing with FSGS-associated variants in the CC domain (33). However, the pathological mechanism of FSGS-associated variants in the ARD and area ii (*e.g.,* G757D and L395A) had not been determined. Our results suggest the ARD and area ii variants also lack sensitivity to [Ca^2+^]_i_, and the resultant disruption of CDI is the likely cause of the sustained cation influx. One remaining question is how the FSGS-associated variants G757D and L395A exert their effects with no clear effect on the peak or integrated current density? We previously showed that open probability and the integrated current density are limited by PLC-catalyzed PIP_2_ depletion (6, 57). Therefore, the contribution of PIP_2_ regulation to the pathophysiology of the FSGS-associated variants should be considered in further studies.

### Impact of delayed inactivation of endogenous TRPC6 in MPC-5 cells

In past studies, WT or FSGS-associated TRPC6 variants were exogenously/endogenously incorporated into mouse podocytes (58, 59, 60). These mice, however, showed only minimal albuminuria and limited changes in glomerular area and podocyte numbers. To confirm the critical contribution of TRPC6 inactivation, we established C6_Δ_CC cells, which exhibit a gain-of-function phenotype manifested as elevated basal [Ca^2+^]_i_ levels as compared to WT and C6K/O cells (Fig. 5, *A* and *B*). The reason for the elevated basal [Ca^2+^]_i_ might be exposure to the mechanical stress in C6_Δ_CC cells. Because the TRPC6 channel is susceptible to mechanical stress (61), the Ca^2+^ influx can be enhanced by the mechanical stimuli in podocytes without stimulation *via* receptor agonists. Furthermore, because TRPC6 is a nonspecific cation channel, aberrant cationic influxes may exert toxic effects in podocytes by modifying the membrane potential and/or signaling pathways (62, 63, 64). Other Ca^2+^ channels, such as Orai channels, may also contribute to the sustained [Ca^2+^]_i_ elevation during FSGS progression (50, 51). Determining which signal pathways are influenced by the sustained Ca^2+^ influx, which is caused by the delayed TRPC6 inactivation, will require further study. For that purpose, C6_Δ_CC cells could be used for drug screening or evaluation based on effects on basal [Ca^2+^]_i_, cell morphology, and expression of podocyte-specific proteins.

### Onset age of FSGS and delayed inactivation in TRPC6

Because the R175Q variant associated with adult-onset and the R175W variant associated with infant-onset FSGS had markedly different profiles in both whole-cell and i/o patch recordings, we speculated that the degree of inactivation and amplification of the peak current density might correlate with the onset of FSGS. To test that hypothesis, the integrated current densities from FSGS-associated TRPC6 variants recorded in whole-cell recordings were plotted against the ages diagnosed with FSGS. Most of the area i variants associated with childhood onset (diagnosed less than 10 years old) showed higher integrated current densities than the WT channel (Fig. 7*A*). To obtain the strongest correlation, we separately compared partial integrated current densities at 0 to 10 s, 30 to 40 s, and 50 to 60 s with peak current density (Fig. 7, *C* and *D*). As shown in Figure 7*D*, the integrated current density from 50 to 60 s exhibited the strongest correlation with the age diagnosed with FSGS (Spearman’s rank correlation coefficient *r*_s_ = 0.85). Nonlinear regression analysis was then applied to the plots, and the fitting equation was as follows,

(1) $$\int_{50}^{60}I\left(\frac{kC}{F}\right)=\alpha \;\ln\left(Years\right)+\beta$$

where *I* is the current density; *C* and *F* are coulombs and farads, respectively; *α* and *β* are slope factors (49.3 for this case) and the integrated current density from 50 to 60 s at 1 year of age (−494.3 *k*C/F). Years is the age diagnosed with FSGS. This equation can be rewritten as:

(2) $$Years=\exp(\left(\int_{50}^{60}I(\frac{kC}{F})+494.3)/49.3\right)$$

For the childhood variants, this equation enables us to speculate the age at FSGS presentation, even for uncharacterized variants (Fig 7*D*).

In summary, we have demonstrated the common features of FSGS-associated TRPC6 variants and the association between dysregulation of this channel and the age at which FSGS first presents. By increasing both basal and stimulated [Ca^2+^]_i_, disrupting the negative regulation of TRPC6 may adversely impact podocyte morphology and differentiation. These findings provide insight into FSGS-associated TRPC6 variants and delayed current inactivation, enabling the identification of disease-causing variants as well as the prediction of the age at disease onset. We believe that such quantitative evaluation of TRPC6 channel activity could be of critical benefit when considering the therapeutic strategy for the treatment of FSGS.

## Experimental procedures

### Molecular biology

All TRPC6 channels were constructed from a plasmid encoding human TRPC6 (GenBank accession no. NM_004621 provided by Dr T. Hofmann) in pIRESn vector, which was modified from the vector pIRES2 (Invitrogen) *via* deletion of the region from IRES to EGFP coding (6). Mutations in human TRPC6 and mouse TRPC6, the latter of which was in pCI-neo expression vector, were generated by the overlap extension and the quick-change PCR utilizing mutagenic primers, respectively (Fig. S5). AT1R encoded plasmid (pcDNA5/FRT/TO-AT1R-APEX) was a gift from Dr A Kruse (Addgene# 96847; http://n2t.net/addgene:96847).

### Cell cultures

HEK293 cells were obtained from ATCC and maintained in DMEM (Gibco) supplemented with 10% FBS (Sigma) and penicillin/streptomycin (Nacalai), at 37 °C and 5% CO_2_. Mouse podocyte clone 5 (MPC-5) cells (provided by Dr Mundel (65)) were conditionally immortalized and cultured at 33 °C in RPMI-1640 medium (Nacalai) with 10% FBS, penicillin/streptomycin, and 50 U/ml recombinant mouse IFN-γ (Wako) during the initial culture. This was replaced with 10 U/ml IFN-γ thereafter until the cell number was sufficient for maturation. To permit podocyte-like cell maturation, temperature in the incubator was switched to 37 °C, and IFN-γ was omitted from the culture medium. For transfection in HEK293 cells, the cells were cotransfected with 0.5 μg each of plasmids encoding wild-type TRPC6 and FSGS-associated variants together with M_1_R and 0.3 μg of plasmids encoding YFP by mixing with superFect transfection reagent (QIAGEN).

### Electrophysiology

Ionic current signals were recorded with a low-noise patch-clamp amplifier (AxoPatch 200B; Axon Instruments). For patch-clamp recording in HEK293 cells, the internal solutions contained in mM: 120 CsOH, 120 aspartate, 20 CsCl, 2 MgCl_2_, 1 EGTA, 0.3 CaCl_2_, 2 ATP-Na_2_, 0.1 GTP, 10 HEPES, and 10 glucose, pH 7.2 (adjusted with Tris-Base), approximately 290 mOSm. Fire-polished patch pipettes had <10 MΩ resistance when backfilled with the internal solution. The external solution contained in mM: 140 NaCl, 5 KCl, 1.8 CaCl_2_, 1.2 MgCl_2_, 10 HEPES, and 10 glucose, 0.1 4,4′-Diisothiocyanatostilbene-2,2′-disulfonic acid (DIDS; Cayman Chemical), pH 7.4, 300 mOsm (adjusted with glucose). Currents were evoked by applying 100 μM carbamylcholine chloride (carbachol; Wako) or 1.0 μM Angiotensin II (PEPTIDE Institute). The currents were recorded at a holding potential of −50 mV. At the end of the recording, N-methyl-d-glucamine (NMDG, 140 mM) containing external solution was applied to confirm cation currents. For i/o recordings, the external solution contained in mM: 120 CsOH, 120 aspartate, 20 CsCl, 2 MgSO_4_, 0 to 3 CaCl_2_, 2 EGTA, 2 HEDTA, 10 HEPES, 10 glucose, 2 ATP-Na_2_, 0.1 GTP, and 0.001 CaM protein, pH 7.2 adjusted with Tris-base (Here and throughout). Free Ca^2+^ concentrations in the solutions were calculated using MaxChelator software (https://maxchelator.stanford.edu). The pipette solution contained in mM: 140 NaCl, 5 KCl, 0.1 CaCl_2_, 2 MgSO_4_, 10 HEPES, 10 glucose, and 0.0003 CCh, pH 7.4. Data were sampled at 20 kHz and filtered at 5 kHz (−3 dB, 4-pole Bessel). During the i/o experiment, the patch membrane was clamped at +70 mV. For whole cell recording in MPC-5 cells, solutions with the following composition were used. The external (mM): 130 sodium gluconate, 0.1 CaCl_2_, 2 MgSO_4_, 10 HEPES, 20 mannitol, 10 Glucose, 0.1 DIDS, and 0.1 N-phenylanthranilic acid (NPA; Cayman Chemical), pH 7.4. The internal: 130 CsOH, 130 glutamate, 2 MgSO_4_, 5 EGTA, 0.5 CaCl_2_, 2 ATP-Na_2_, 0.1 GTP, 10 HEPES, and 10 glucose, pH 7.2. Cells were continuously perfused with a gravity-fed external solution at a flow rate of 0.5 ml/min. The perfusion is turned on and off using electromagnetic solenoid microvalves (Takasago).

### TRPC6 gene-edited MPC-5 generated by CRISPR/Cas9 system

The targets sequence of mouse TRPC6 (Fig. S6) was subcloned as a gRNA target sequence for gene editing using CRISPR-Cas9 into the pGuide-it-ZsGreen1 vector (Clontech) following the manufacturer’s protocol. Transfection of pGuide-it-ZsGreen1 construct into immature MPC-5 was achieved by using Lipofectamine 3000 (Invitrogen). A bright green fluorescent protein, ZsGreen1, expressed clones were isolated by BD FACS Melody cell sorter (BD Biosciences). After single-cell isolation, genomic DNA was extracted using the Guide-it Mutation Detection Kit (Clontech). The sequence from 135,028 to 136,141 in *TRPC6* gene (NCBI Gene ID: 22068) was confirmed with the specific primers.

### Cell surface biotinylation and simple western assay

MPC-5 cells (2.2 × 10^4^ cells/cm^2^) were incubated for 2 weeks at 37 °C, and counted before the treatment. The culture dishes were incubated with 0.5 mg/ml Sulfo-NHS-SS-Biotin (Thermo Fisher Scientific) for 30 min at R.T. After washing with 100 mM glycine in PBS, the cells were then lysed with RIPA buffer. The concentrations of supernatants were adjusted with each cell number, and the sample was added to streptavidin-agarose beads (Thermo Fisher Scientific). The biotinylated membrane fraction was eluted with 1 × SDS sample buffer. Eluates were analyzed as membrane fractions by the Wes Simple Western instrument (ProteinSimple). For the operation of Simple Western, we followed the manufacturer’s standard method for the 12 to 230-kDa separation module. Anti-TRPC6 antibody (Alomone) was diluted 20-fold with Canget Signal 2 (Toyobo). A digital image of the chemiluminescence of the capillary was captured with Compass Simple Western software. An internal system control was included in each run.

### Calcium imaging of MPC-5 cell lines

Ca^2+^ imaging analysis with Fura-2 for MPC-5 cells was performed as reported previously (33).

### Measurement of single-cell area

C6_Δ_CC cells were cultured for 3 to 4 days at 37 °C and seeded on glass coverslips placed on 12-well plates at a density of 2 × 10^3^ cells per well. Cells were cultured with 1 nM-100 nM SAR-7334 and were incubated for 24 h. To measure single cell area, the cells were stained with 1 μM calcein-AM (Nacalai Tesque) and 1 μg/ml Hoechst 33342 (Dojindo) for 10 min, and captured images using a fluorescence microscope (BZ-X800, Keyence). The fluorescence area divided by the number of cells was defined as the single cell area in this study. The signals were quantified by Image J software (NIH). For measurement of synaptopodin expression, the cells were stained with anti-synaptopodin antibody with the following protocol.

### Immunofluorescence staining

MPC-5 cells were grown on glass coverslips and were fixed with 4% paraformaldehyde (PFA). The cells were blocked and permeabilized with 0.5% Triton X-100/5% bovine serum albumin for 30 min. Then rabbit polyclonal anti-TRPC6 antibody (1:50, Alomone) and mouse monoclonal anti-nephrin antibody (1:50, Santa Cruz Biothecnology, sc-377246), or mouse monoclonal anti-synaptopodin antibody (1:2, Progen, clone G1D4) were incubate for overnight. The following day, cells were applied Alexa Fluor 488 anti-mouse IgG (Abcam, 1:1000) or CF594 anti-rabbit IgG (Biotium, 1:2000) for 1 h at room temperature with DAPI nuclear stain (Dojindo). Image capturing was performed with fluorescence microscope (BZX-800, Keyence).

### Phalloidin staining

The F-actin structures in MPC-5 cells were confirmed as described previously (66).

### RNA sequencing

RNA was extracted from MPC-5 cells using the RNeasy mini kit (QIAGEN) and was confirmed to be over 7.0 RIN using Bioanalyzer (Agilent). Poly (A) RNA was extracted from 100 ng of total RNA with NEBNext poly(A) mRNA magnetic isolation module (NEB). The library was prepared according to the instruction manual of the NEBNext ultra Ⅱ RNA library prep kit (NEB). Briefly, cDNA was synthesized and amplified by PCR to prepare a library labeled with a barcode sequence to identify the sample. The library was analyzed using a NovaSeq 6000 system (Illumina). The gene reads were mapped to the reference sequence following trimming, and tag counts were performed. The number of transcript reads corresponding to each gene ID and transcript ID was counted using the RNA-Seq Analysis tool (CLC Genomics Workbench), and normalized values (TPM, transcripts per million) were calculated based on the gene length and read count.

### Statistical analysis

Data are expressed as the mean ± SD. Statistical significance was evaluated by Student’s *t* test using Jamovi software (version 2.2; retrieved from https://www.jamovi.org) or one-way ANOVA with Tukey’s *post hoc* comparison tests. The Dunnett multiple comparison tests comparing each group to control (wild-type) were examined by using EZR (easy R) software (version 1.67) (67). *p* < 0.05 was considered as a significant difference.

## Data availability

All data generated or analyzed during this study are available from the corresponding author on reasonable request.

## Supporting information

This article contains supporting information (14, 15, 16, 17, 20, 21, 23, 26, 27, 29, 30, 32, 68).

## Conflict of interest

The authors declare that they have no conflicts of interest with the contents of this article.
