# Supplementary material for: Delayed inactivation of TRPC6 as a determinative characteristic of FSGS-associated variants
Source: J Biol Chem. 2025 May 21;301(6):110256. doi: 10.1016/j.jbc.2025.110256 (PMC12206031; doi:10.1016/j.jbc.2025.110256)
Supplement: Supplementary Figures [file mmc1.docx]

**Supporting information for**

**“Delayed inactivation of TRPC6 as a determinative characteristic of FSGS-associated variants”**

**Figure S1. TRPC6 variants and age at FSGS diagnosis**

**Figure S2. Comparison of expression levels of FSGS-associated TRPC6 variants**.

**Figure S3. Effects of SAR-7334 on expression of synaptopodin in C6_Δ_CC cells**.

**Figure S4. Transcripts per million (TPM) of the podocyte-specific essential genes in WT, C6K/O and C6_Δ_CC cells.**

**Figure S5. Primers for the constructions.**

**Figure S6. Oligo DNA for the construction with pGuide-it Vector**.

**Figure S7. Uncropped Simple Western data depicting TRPC6 expression levels in podocytes.**

**Figure S1. TRPC6 variants and age at FSGS diagnosis**

| Variants | Protein change | Protein domain | Area | Age (year) | Reference |
| --- | --- | --- | --- | --- | --- |
| c.325G>A | G109S | ARD | (i) | 21 | (1) |
| c.326 G > A | G109D | ARD | (i) | 8 | (2) |
| c.335C>A | P112Q | ARD | (i) | 30 | (3) |
| c.389 T>A | D130V | ARD | (i) | 5 | (4) |
| c.517 T > G | Y173D | ARD | (i) | 3 | (2) |
| c.524G>A | R175Q | ARD | (i) | 27,53 | (5) |
| c.523C>T | R175W | ARD | (i) | 0.3, 2 | (2, 6) |
| c.653A>T | H218L | ARD | (i) | 8 | (7) |
| c.808C>A | S270T |  | (i) | 20, 20, 22, 52 | (8, 9) |
| * | L395A |  | (ii) | 2.4 | (10) |
| c.2270G>A | G757D |  | (ii) | 1 | (11, 12) |
| c.2339 T>C | L780P |  | (ii) | 7 | (1) |
|  | K874X |  | (i) | 27, 55, 57 | (8, 9) |
| c.2684G>T | R895L | coiled-coil | (i) | 2 | (7) |
| c.2683C>T | R895C | coiled-coil | (i) | 3, 5, 7, 18, 21, 30, 35, 46 | (2, 8) |

* The sequence of L395A variant was not described in the reference.

**
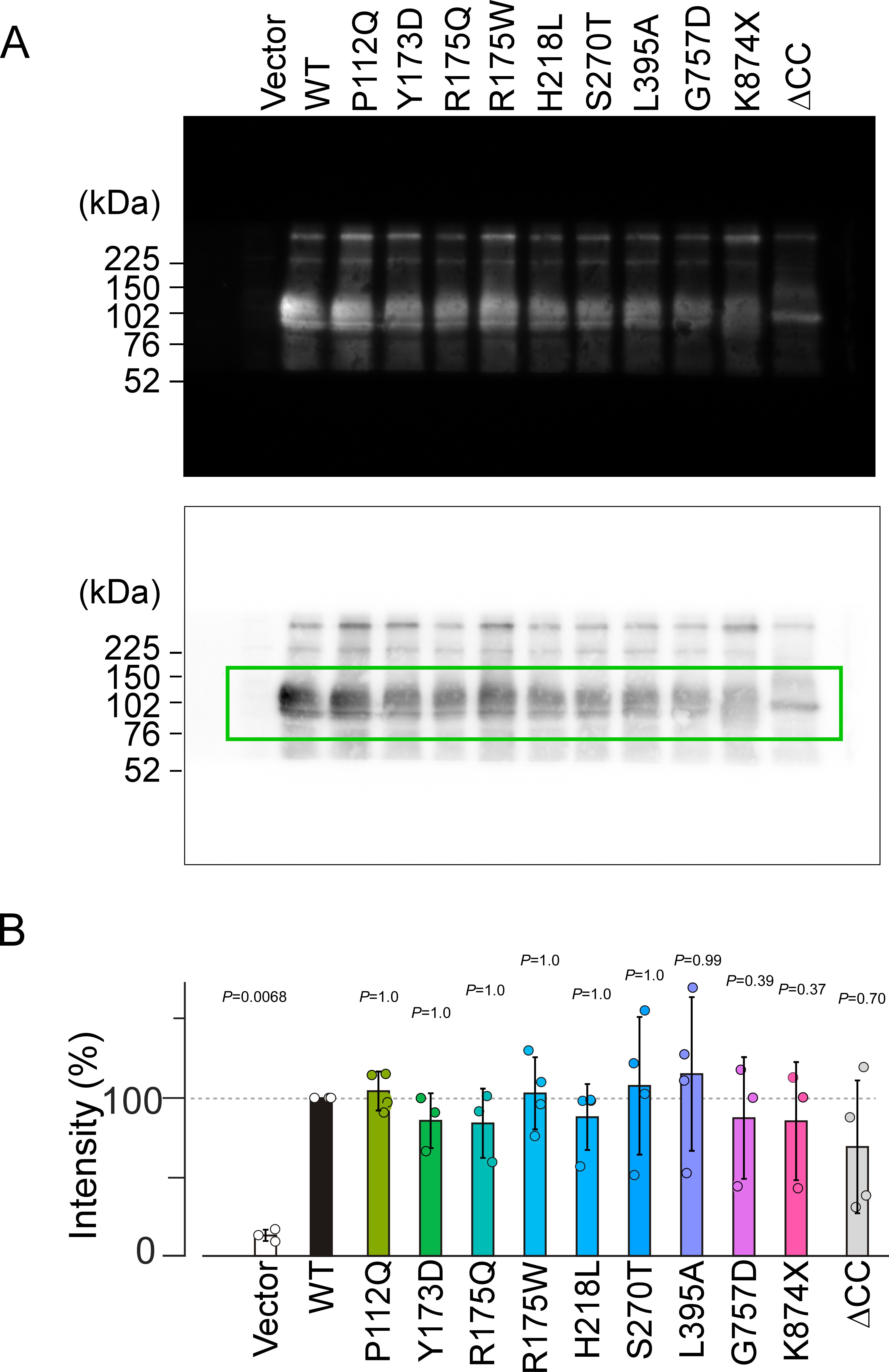
**

**Figure S2. Comparison of expression levels of FSGS-associated TRPC6 variants.**

(A) Detection of TRPC6 expressed in HEK293 cells by 7.5% SDS-PAGE and western blotting with the anti-TRPC6 antibody. FSGS variants displayed either increased peak current density (P112Q, Y173D, R175Q, R175W, H218L, K874X in Figure 2D) or no statistical differences (S270T, L395A, G757D) were examined in this blotting. Chemiluminescence (upper) and the inverted (lower) images, respectively. The green square areas were subjected to the analysis. (B) The statistical analysis of the western blotting. The respective signals were normalized by the WT signal in each experiment. Individual data points indicated technical replicates (3-4). The columns and bars are mean ± SD, respectively. *p*-values are determined by one-way ANOVA with Dunnett’s *post hoc* test.

**Methods for TRPC6 expression levels in HEK cells**

All TRPC6 proteins were transiently expressed in HEK293 cell. At 48 hours after transfection, the cells were lysed in RIPA buffer and extracts were centrifuged at 20,400x g for 10 min. The supernatants containing 13 μg of total proteins were then resolved using 7.5% SDS-PAGE. The proteins in the gel were electro-transferred to a PVDF membrane (Cytiva, pore size 0.2 *μ*m) with constant current at 0.002 A/cm^2^ for 30 min. The membrane was incubated in blocking buffer (25 mM Tris, 150 mM NaCl, pH 7.5, 0.1% Tween 20 [TBST] with 0.3% skimmed milk) for 1 hour at RT and was incubated overnight at 4°C with rabbit anti-TRPC6 (cat. no. ACC-017, Alomone, 1:200 dilution with Can Get Signal solution 1 provided by Toyobo). After treatment with the primary antibody, the membrane was washed thrice with TBST for 5 minutes each time. The secondary antibody (anti-rabbit IgG, HRP conjugated; Cytiva, 1:2000 dilution with Can Get Signal solution 2) was applied onto the membrane for 1 hour at RT. The bands were detected by chemiluminescence (ECL Prime Western blotting Detection Reagent; Cytiva). An imager equipped with CCD was used for data analysis (Light-Capture; ATTO).

**
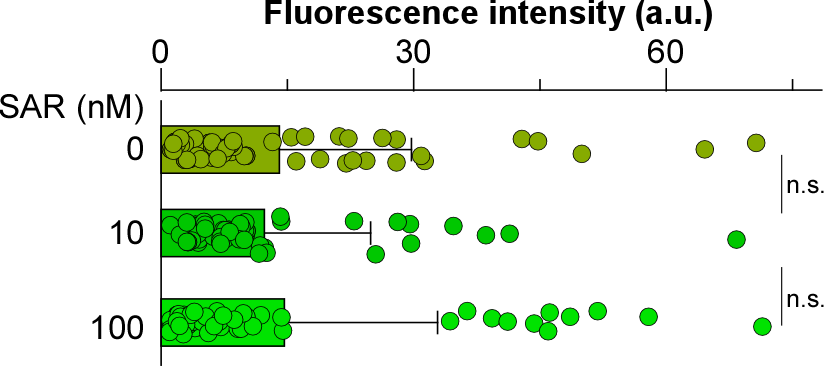
**

**Figure S3. Effects of SAR-7334 on expression of synaptopodin in C6_Δ_CC cells.**

A bar graph showing quantification of fluorescence intensity of SAR-7334 treated C6_Δ_CC cells. More than 50 cells were analyzed for each SAR-7334 treatment condition. Populations with robust signal cells were increased by treatment of SAR-7334 (100 nM). We thus analyze the cells exceeding the threshold fluorescent intensity (15 arbitrary unit), which is shown in Figure 6J. The column and bars represent the mean and SD, respectively. Individual data points the fluorescence intensity of each cell. *P* values were calculated using one-way ANOVA with Tukey’s *post hoc* test.

**Figure S4. Transcripts per million (TPM) of the podocyte-specific essential genes in WT, C6K/O and C6_Δ_CC cells**.

| Gene symbols | WT | C6_Δ_CC | C6K/O |
| --- | --- | --- | --- |
| Aif1l | 132.0 | 117.0 | 126.6 |
| Alcam | 32.29 | 7.504 | 3.483 |
| Anxa4 | 50.06 | 65.82 | 61.09 |
| Aox1 | 3.994 | 2.599 | 3.206 |
| Arhgap24 | 30.52 | 36.92 | 28.80 |
| Arhgef18 | 29.90 | 20.01 | 17.40 |
| Arpc1a | 110.1 | 187.0 | 89.39 |
| Cd59a | 96.38 | 34.90 | 78.29 |
| Cdkn1c | 7.667 | 0 | 10.51 |
| Cers6 | 36.27 | 22.75 | 28.49 |
| Clic3 | 0.3089 | 0.6221 | 0.4429 |
| Clic5 | 0.3072 | 0.1302 | 0.2936 |
| Col4a3 | 17.16 | 0.8844 | 0.4197 |
| Cpa6 | 0.02285 | 0.04602 | 0.06553 |
| Cryab | 2311 | 1343 | 840.0 |
| Ctsl | 335.6 | 196.3 | 119.1 |
| Cyb5r4 | 28.79 | 29.37 | 18.80 |
| D330041h03RIK | 1.810 | 4.128 | 10.00 |
| Dnajc11 | 18.76 | 23.07 | 24.60 |
| Dpp4 | 0.02474 | 0 | 0.02364 |
| Dtnb | 30.07 | 32.40 | 24.99 |
| Enpep | 4.700 | 11.79 | 38.10 |
| Epb41l5 | 9.051 | 11.66 | 8.169 |
| Ezr | 1015 | 755.2 | 655.4 |
| Fgfr1 | 28.68 | 18.51 | 10.61 |
| Fnbp1l | 50.71 | 66.40 | 57.13 |
| Foxd2os | 5.7 | 10.28 | 9.8 |
| Gadd45a | 74.09 | 166.9 | 310.2 |
| Gm13589 | 0.04666 | 0 | 0 |
| Gm4117 | 0.1136 | 0.03267 | 0.1099 |
| Golim4 | 57.44 | 31.13 | 48.63 |
| Gpc1 | 211.8 | 144.8 | 71.89 |
| H2-Q7 | 88.85 | 222.9 | 392.0 |
| Haus8 | 15.02 | 25.80 | 30.44 |
| Ift80 | 13.93 | 24.43 | 58.20 |
| Ildr2 | 0.02842 | 0 | 0.1267 |
| Iqgap2 | 0.04525 | 0.1367 | 0.1946 |
| Itgav | 196.7 | 90.91 | 84.42 |
| Itgb5 | 593.4 | 374.5 | 257.1 |
| Kank1 | 44.77 | 74.05 | 19.71 |
| Lgr4 | 41.35 | 51.94 | 33.68 |
| Lpin2 | 19.80 | 14.94 | 13.04 |
| Lpl | 0.07460 | 0 | 0.07129 |
| Mafb | 16.04 | 12.10 | 3.376 |
| Magi2 | 0.9161 | 0.7906 | 3.074 |
| Mapt | 2.065 | 15.34 | 1.597 |
| Mocs2 | 17.91 | 18.10 | 18.84 |
| mt-Atp8 | 2569 | 2001 | 3238 |
| Mtss1 | 5.587 | 6.216 | 4.635 |
| Myom2 | 0.01766 | 0 | 0 |
| Nebl | 0 | 0.02066 | 0.02452 |
| Nphs1 | 0.2007 | 0.2021 | 0.2934 |
| Npnt | 195.3 | 126.7 | 56.06 |
| Nsf | 15.12 | 17.42 | 17.98 |
| Nupr1 | 126.5 | 111.9 | 146.6 |
| Pak1 | 4.460 | 1.297 | 9.188 |
| Pals1(Mpp5) | 49.89 | 43.59 | 34.44 |
| Parva | 110.7 | 95.20 | 80.83 |
| Plce1 | 92.22 | 20.58 | 16.98 |
| Podxl | 10.35 | 18.83 | 1.116 |
| Psg16 | 0 | 0 | 0.08167 |
| Ptpro | 0.02325 | 0.04682 | 0.04444 |
| Rab3b | 64.41 | 20.32 | 6.147 |
| Robo2 | 2.435 | 1.539 | 0.1759 |
| Rpl37(Rpl37rt) | 590.0 | 772.9 | 645.8 |
| Schip1 | 54.65 | 39.55 | 58.75 |
| Sdc4 | 584.3 | 606.1 | 637.4 |
| Sema3g | 7.885 | 12.33 | 13.90 |
| Septin10(Sept10) | 40.04 | 59.92 | 92.28 |
| Shisa3 | 0 | 0 | 0.06285 |
| Srgap1 | 4.759 | 2.465 | 1.399 |
| Synpo | 197.9 | 157.1 | 222.1 |
| Tdrd5 | 0.7324 | 0.4538 | 0.5599 |
| Thsd7a | 0.9900 | 0.6932 | 0.3535 |
| Tmod3 | 82.38 | 81.48 | 86.76 |
| Tsc22d1 | 242.9 | 264.6 | 186.9 |
| Vegfa | 76.01 | 63.20 | 65.29 |
| Wt1 | 0.5551 | 0.4065 | 0.09646 |

In 92 podocyte-specific essential genes summarized by Lu et al. (13), the TPM values of 78 genes which were detected by the RNA-sequence analysis were listed.

**Figure S5. Primers for the constructions**.

| Constructs/Vector | Forward 5’-3’ | Reversal 5’-3’ |
| --- | --- | --- |
| hTRPC6_G109S/pIRESn | GCAGCTGAATATTCTAACATCCCAG | CTGGGATGTTAGAATATTCAGCTGC |
| hTRPC6_G109D/pIRESn | GCTGAATATGATAACATCCCAGTGGTGC | GCACCACTGGGATGTTATCATATTCAGC |
| hTRPC6_P112Q/pIRESn | GGTAACATCCAAGTGGTGCGGA | TCCGCACCACTTGGATGTTACC |
| hTRPC6_D130V/pIRESn | AACTGTGTGGTTTACATGGGC | GCCCATGTAAACCACACAGTT |
| hTRPC6_Y173D/pIRESn | TAGTAAAGGTGATGTTCGGATTG | CAATCCGAACATCACCTTTACTA |
| hTRPC6_R175Q/pIRESn | GGTTATGTTCAGATTGTGGAAGC | GCTTCCACAATCTGAACATAACC |
| hTRPC6_R175W/pIRESn | GGTTATGTTTGGATTGTGGAAGC | GCTTCCACAATCCAAACATAACC |
| hTRPC6_H218L/pIRESn | CGGTTCTCCCTTGATGTGACTC | GAGTCACATCAAGGGAGAACCG |
| hTRPC6_S270T/pIRESn | TTTAGCCACACCAGATCTAG | CTAGATCTGGTGTGGCTAAA |
| hTRPC6_L395A/pIRESn | GGTATGAGAATGCTTCTGGTTTACG | CGTAAACCAGAAGCATTCTCATACC |
| hTRPC6_G757D/pIRESn | TTTGAGGAGGACAGAACACTT | AAGTGTTCTGTCCTCCTCAAA |
| hTRPC6_L780P/pIRESn | CTTACTGAAGCCTAAAAAATGGATTTC | GAAATCCATTTTTTAGGCTTCAGTAAG |
| hTRPC6_K874X/pIRESn | GCCCAGATAGATTAGGAGAGTGATG | CATCACTCTCCTAATCTATCTGGGC |
| hTRPC6_R895L/pIRESn | GACATCTCAAGTCTCCTCTATGAACTCC | GGAGTTCATAGAGGAGACTTGAGATGTC |
| hTRPC6_R895C/pIRESn | CATCTCAAGTCTCTGCTATGAACTCC | GGAGTTCATAGCAGAGACTTGAGATG |

**Figure S6. Oligo DNA for the construction with pGuide-it Vector**.

mTRPC6ΔCC

• Oligo 1: 5’-ccggGATTGATAAGGAGAGCGATG -3’

• Oligo 2: 5’-aaacCATCGCTCTCCTTATCAATC-3’

mTRPC6K/O

• Oligo 1: 5’-ccggAGGTTATGTACGGATTGTGG -3’

• Oligo 2: 5’-aaacCCACAATCCGTACATAACCT-3’


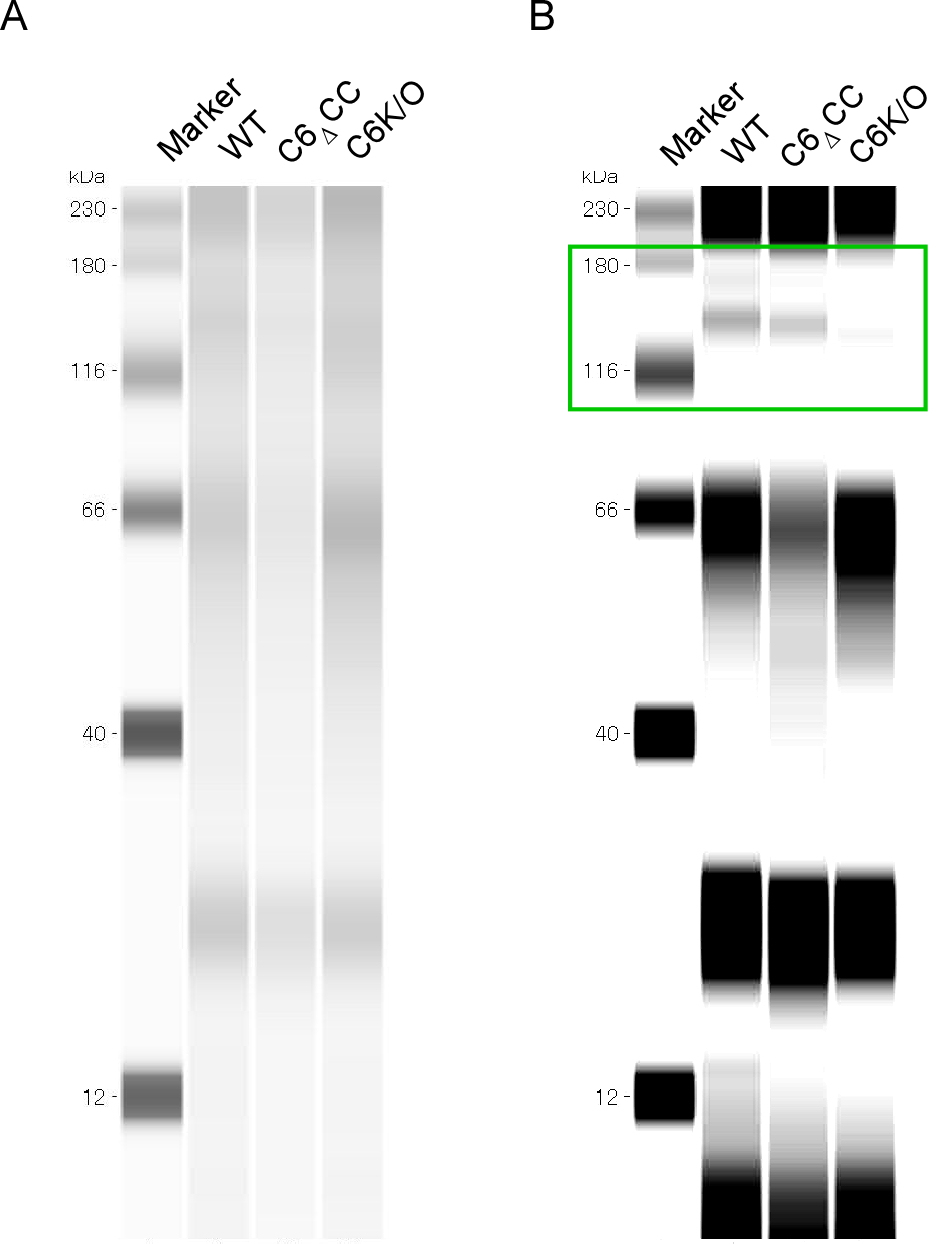


**Figure S7. Uncropped Simple Western data depicting TRPC6 expression levels in podocytes.**

Raw data (A) and uncropped digitized data (B) corresponding to Figure 5F in the main manuscript. The green square indicates the cropped area.

**Supplemental references**

1. Santin, S., Ars, E., Rossetti, S., Salido, E., Silva, I., Garcia-Maset, R. *et al.* (2009) TRPC6 mutational analysis in a large cohort of patients with focal segmental glomerulosclerosis Nephrol Dial Transplant **24**, 3089-3096 10.1093/ndt/gfp229

2. Nagano, C., Yamamura, T., Horinouchi, T., Aoto, Y., Ishiko, S., Sakakibara, N. *et al.* (2020) Comprehensive genetic diagnosis of Japanese patients with severe proteinuria Sci Rep **10**, 270 10.1038/s41598-019-57149-5

3. Winn, M. P., Conlon, P. J., Lynn, K. L., Farrington, M. K., Creazzo, T., Hawkins, A. F. *et al.* (2005) A mutation in the TRPC6 cation channel causes familial focal segmental glomerulosclerosis Science **308**, 1801-1804 10.1126/science.1106215

4. Gheissari, A., Meamar, R., Kheirollahi, M., Rouigari, M., Dehbashi, M., Dehghani, L. *et al.* (2018) TRPC6 Mutational Analysis in Iranian Children With Focal Segmental Glomerulosclerosis Iran J Kidney Dis **12**, 341-349, <https://www.ncbi.nlm.nih.gov/pubmed/30595563>

5. Hofstra, J. M., Lainez, S., van Kuijk, W. H., Schoots, J., Baltissen, M. P., Hoefsloot, L. H. *et al.* (2013) New TRPC6 gain-of-function mutation in a non-consanguineous Dutch family with late-onset focal segmental glomerulosclerosis Nephrol Dial Transplant **28**, 1830-1838 10.1093/ndt/gfs572

6. Wang, F., Zhang, Y., Mao, J., Yu, Z., Yi, Z., Yu, L. *et al.* (2017) Spectrum of mutations in Chinese children with steroid-resistant nephrotic syndrome Pediatr Nephrol **32**, 1181-1192 10.1007/s00467-017-3590-y

7. Gigante, M., Caridi, G., Montemurno, E., Soccio, M., d'Apolito, M., Cerullo, G. *et al.* (2011) TRPC6 mutations in children with steroid-resistant nephrotic syndrome and atypical phenotype Clin J Am Soc Nephrol **6**, 1626-1634 10.2215/CJN.07830910

8. Reiser, J., Polu, K. R., Moller, C. C., Kenlan, P., Altintas, M. M., Wei, C. *et al.* (2005) TRPC6 is a glomerular slit diaphragm-associated channel required for normal renal function Nat Genet **37**, 739-744 10.1038/ng1592

9. Heeringa, S. F., Moller, C. C., Du, J., Yue, L., Hinkes, B., Chernin, G. *et al.* (2009) A novel TRPC6 mutation that causes childhood FSGS PLoS One **4**, e7771 10.1371/journal.pone.0007771

10. Mir, S., Yavascan, O., Berdeli, A., andSozeri, B. (2012) TRPC6 gene variants in Turkish children with steroid-resistant nephrotic syndrome Nephrol Dial Transplant **27**, 205-209 10.1093/ndt/gfr202

11. Büscher, A. K., Kranz, B., Buscher, R., Hildebrandt, F., Dworniczak, B., Pennekamp, P. *et al.* (2010) Immunosuppression and renal outcome in congenital and pediatric steroid-resistant nephrotic syndrome Clin J Am Soc Nephrol **5**, 2075-2084 10.2215/CJN.01190210

12. Riehle, M., Buscher, A. K., Gohlke, B. O., Kassmann, M., Kolatsi-Joannou, M., Brasen, J. H. *et al.* (2016) TRPC6 G757D Loss-of-Function Mutation Associates with FSGS J Am Soc Nephrol **27**, 2771-2783 10.1681/ASN.2015030318

13. Lu, Y., Ye, Y., Bao, W., Yang, Q., Wang, J., Liu, Z. *et al.* (2017) Genome-wide identification of genes essential for podocyte cytoskeletons based on single-cell RNA sequencing Kidney Int **92**, 1119-1129 10.1016/j.kint.2017.04.022
